# Modifiable Factors Influencing Disease Flares in Inflammatory Bowel Disease: A Literature Overview of Lifestyle, Psychological, and Environmental Risk Factors

**DOI:** 10.3390/jcm14072296

**Published:** 2025-03-27

**Authors:** Lola J. M. Koppelman, Aroha A. Oyugi, P. W. Jeroen Maljaars, Andrea E. van der Meulen-de Jong

**Affiliations:** Department of Gastroenterology and Hepatology, Leiden University Medical Centre, 2333 ZA Leiden, The Netherlands

**Keywords:** inflammatory bowel disease, disease flares, risk factors, nutrition, psychological health, sleep quality, predictive models, lifestyle factors, personalized treatment

## Abstract

**Background:** A significant concern for patients with Inflammatory Bowel Disease (IBD) is predicting and managing disease flares. While healthcare providers rely on biomarkers, providing conclusive patient advice remains challenging. This review explores the role of lifestyle, psychological health, and environmental exposures in the prediction and management of IBD flares. **Methods:** This review followed PRISMA guidelines (2020). A structured search was conducted in PubMed for articles published between 2012 and 2024, using free and Medical Subject Heading (MeSH) terms for predicting factors in IBD. Inclusion criteria included studies reporting primary data on modifiable clinical or environmental predictors of IBD relapse, excluding studies on post-operative investigations, treatment cessation, and pediatric or pregnant populations. The Mixed Method Appraisal Tool (MMAT) was used to assess the quality of the studies. **Results:** Out of 2287 identified citations, 58 articles were included. Several modifiable factors influencing disease flares were identified, including psychological stress, sleep disturbances, smoking, and nutrition. Poor sleep quality and mental health were linked to increased flare risks, while smoking was associated with higher relapse rates in Crohn’s disease. Environmental exposures, such as heat waves and high-altitude regions, also contributed. Predictive models integrating clinical, lifestyle, and psychological factors showed promising accuracy but require further refinement. Limitations of this review include the potential for publication bias, variability in flare definitions, and limited sample sizes **Conclusions:** Key predictors of IBD flares include dietary factors, psychological stress, poor sleep quality, and pharmacological influences. Personalized approaches integrating these predictors can optimize disease control and improve patient outcomes.

## 1. Introduction

Healthcare providers of patients with inflammatory bowel disease (IBD) are often faced with questions and statements from their patients that fall outside the realm of well-established scientific evidence. Specifically, there is a recurring concern among patients about how to effectively manage their disease [1,2]. For example, patients with Crohn’s disease (CD) may express concerns about reducing their risk of future flares and whether certain activities, such as altitude training, might trigger them. While factors like age at disease onset and medical history of disease activity are well-documented, less attention has been paid to modifiable external and lifestyle factors under the patient’s control, such as diet, stress management, or physical activity. This gap in knowledge often leaves healthcare providers with limited answers to address their patients’ concerns, despite growing evidence that modifiable environmental and behavioral factors may influence disease activity. CD and Ulcerative Colitis (UC), collectively known as IBD, are chronic inflammatory diseases of the gastrointestinal tract. They are characterized by their relapsing nature, alternating between periods of remission and active disease. The incidence of IBD is rising, especially in high-income countries. Its etiology remains largely unknown; however, a complex interplay between genetic, immunological, gut microbiome, and environmental factors is thought to contribute to its onset [3,4,5]. These factors are also believed to influence disease progression and the risk of relapse [6]. For example, studies suggest that dietary patterns, smoking, and chronic psychological stress can contribute to disease flares, but comprehensive guidance on managing these factors in daily life remains limited [7,8,9].

For patients, the unpredictable nature of flares might add a significant burden to the already challenging disease. Symptoms such as severe abdominal pain, diarrhea, fatigue, and weight loss not only affect physical health but also take a toll on psychological well-being. To prevent structural damage and disability, the current treatment goal is to achieve and maintain clinical and biochemical remission with the aim of achieving deep remission and mucosal healing [10,11,12,13,14,15]. However, disease flares remain a major challenge, often arising unexpectedly and worsening the disease course. Early intervention is proposed as a strategy to mitigate these complications, but for many patients, the question remains: “What proactive steps can I take to reduce my risks?” [16,17].

This review describes how external and patient-reported factors contribute to disease flares in IBD, focusing on less explored influences that are often raised from the patients’ perspective. This review aims to provide actionable insights that empower both patients and healthcare providers. By considering these factors, this approach not only complements existing knowledge of well-established risks for a flare but also addresses the critical need for more personalized and patient-centered care in IBD management.

## 2. Materials and Methods

### 2.1. Search Strategy

A structured literature search was conducted in PubMed on 5 March 2024 to synthesize clinical and external predictors of impending relapse in patients with IBD. To identify all eligible studies, both free words and Medical Subject Heading (MeSH) terms for predicting factors of relapse in IBD patients were used. The complete search query is provided in Appendix A. Filters were applied to include studies conducted in humans, in vivo, and published between 2012 and 2024. Articles in English or Dutch were considered. The review methodology adhered to the updated PRISMA guidelines (2020) and was further tailored to address the aim of identifying actionable and patient-centered factors influencing disease flares [18]. This review did not follow a pre-registered protocol.

### 2.2. Selection Process

The articles were independently screened for relevance based on title and abstract by two authors (L.J.M.K. and A.A.O.). For articles that were deemed potentially relevant, the full text was retrieved and assessed. Studies were included if they reported primary data on at least one predictive factor for IBD relapse.

The review focused on actionable and modifiable factors that could inform practical strategies for managing IBD flares. Clinical factors were defined as patient-centered outcomes measurable in clinical practice or through validated instruments, such as quality of life, psychological health, and clinical indices (e.g., patient-reported outcome measures or disease activity scores). External factors were defined as behavioral or environmental exposures modifiable through lifestyle changes or patient education, including smoking, nutrition, environmental exposures, and physical activity. This classification was informed by existing literature on IBD risk factors and was designed to align with the review’s aim of identifying patient-driven, modifiable influences on disease flares.

Established predictors such as systemic comorbidities or biochemical, imaging, and microbiome predictors were excluded as they were outside the scope of this review. Additionally, studies involving treatment cessation, post-operative investigations, and pediatric or pregnant populations were excluded. No restrictions were applied regarding adult age groups or geographical settings of the clinical studies.

### 2.3. Data Extraction and Synthesis

Once the articles passed all stages of screening, the following items were extracted from the studies: author, year of publication, journal, study design, population, intervention, outcome measure, type of predictor, key results, and conclusions. Effect measures (e.g., odds ratios, hazard ratios, and mean differences) were extracted where reported, and, in cases where consistent effect sizes were not available, the results were summarized narratively. A data quality appraisal was performed for each article to evaluate the quality, reliability, and relevance of the data presented in each article. The Mixed Methods Appraisal Tool (MMAT) was used to assess each article [19].

To synthesize the findings, a thematic analysis was conducted. Studies were grouped according to the main predictors or risk factors they addressed, and these were categorized into overarching themes. The results were compared across studies, highlighting both detrimental and protective effects. All steps of data extraction, quality appraisal, and synthesis were performed independently by two authors (L.J.M.K. and A.A.O.). Data from studies of higher quality were given more weight in the synthesis, while studies with lower quality were considered with caution to minimize bias in the overall findings.

## 3. Results

**Figure 1:**
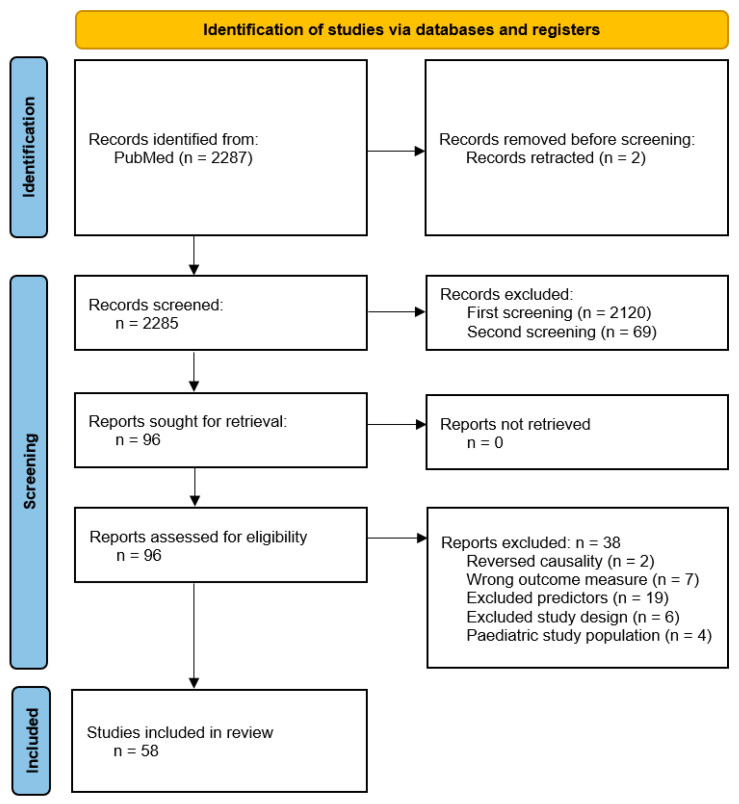
PRISMA 2020 flow diagram.

**Figure 2:**
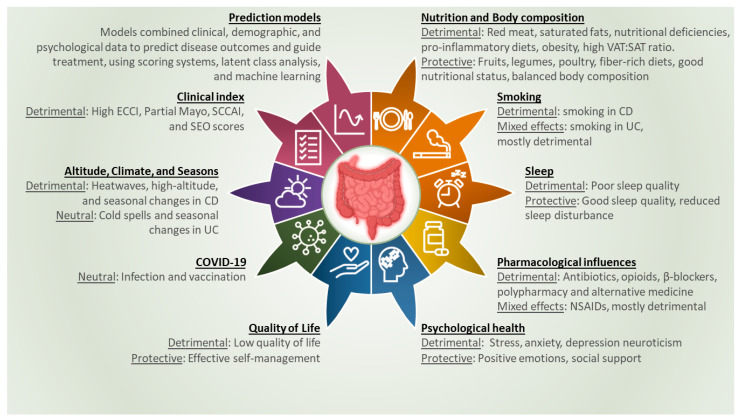
Summary of identified risk factors for disease flares in inflammatory bowel disease. Abbreviations: VAT:SAT, Visceral Adipose Tissue–Subcutaneous Adipose Tissue; CD, Crohn’s Disease; UC, Ulcerative Colitis; NSAIDs, Non-steroidal Anti-inflammatory Drugs; COVID-19, Coronavirus Disease 2019; ECCI, Endoscopic Clinical Correlation Index; SCCAI, Simple Clinical Colitis Activity Index; SEO, Seo’s Activity Index.

The initial search resulted in 2287 citations. Screening based on title and abstract resulted in 96 articles eligible for full-text screening (Figure 1). After full-text assessment, an additional 38 reports were excluded, resulting in 58 articles being included in the final analysis. These studies were categorized based on the main predictors or risk factors they studied, which were grouped into six overarching themes: lifestyle factors, non-IBD medications, psychological and behavioral factors, Coronavirus Disease 2019 (COVID-19), environmental factors, and multifactorial assessments. Several studies emphasized the complex interplay between these factors, suggesting that the accumulation of multiple risk factors may have a detrimental effect on disease flares. Conversely, the presence of protective factors, such as stress management or dietary adjustments, could counteract these effects. Figure 2 provides a structured overview of these key predictors and risk factors, highlighting both detrimental and protective effects. A more detailed summary of the included articles can be found in Table 1.

### 3.1. Quality Assessment

The overall quality of the studies, as assessed using the MMAT, ranged from moderate to good. Common concerns included small sample sizes and short follow-up periods. Most of the included studies were cohort studies, while only three randomized controlled trials were identified in the review. The MMAT scores for all articles used in this review can be found in Appendix B.

### 3.2. Lifestyle and Diet

#### 3.2.1. Nutrition and Body Composition

Nutrition is a common topic of discussion during consultations with IBD patients as it represents an aspect of their health they can actively manage. Diet was evaluated in fourteen studies, and while some common themes emerged, the relationship between nutrition and IBD remains complex. Several studies suggest that specific foods and ingredients may either exacerbate or mitigate disease activity. For instance, Tasson et al. and Mi et al. observed that frequent consumption of red meat, chilis, milk, fish, and nuts increased relapse risk, whereas fruits, legumes, poultry, and potatoes appeared protective [20,21]. However, other studies have reported no significant association between meat intake and disease flares [22,23,24].

In an analysis of specific ingredients and macronutrients, saturated fatty acids—particularly myristic acids found in palm oil, coconut oil, and dairy fats—were linked to an increased risk of flares [23]. Moreover, in a small pilot study (*n* = 17), a low-fat, high-fiber diet reduced inflammation and improved gut microbiota in UC [25]. This aligns with evidence of fiber’s protective role in CD [26]. However, in this same study, researchers also found that fiber intake did not significantly affect flare risk in UC, with some data even suggesting a potential increase in risk. The role of additives further complicates the issue; for example, carrageenan, a common emulsifier, was associated with shorter time to relapse in UC patients in a small, randomized controlled trial (*n* = 12) [27].

When looking at the diet as a whole, researchers found a pro-inflammatory diet, as indicated by a higher Dietary Inflammatory Index, was associated with elevated fecal calprotectin levels, altered gut microbiota, and a higher risk of relapse in IBD patients [28].

Nutritional deficiencies, particularly in zinc and selenium, are another area of concern. Zinc deficiency was associated with an increased need for corticosteroids and surgery in CD, while selenium deficiency was linked to higher relapse rates in UC [29]. In contrast, higher intake of calcium, riboflavin, and vitamin A have been identified as potential triggers for UC relapse [75].

Nutritional status is also a factor in predicting disease flares in IBD. Spooren et al. analyzed data from 417 IBD outpatients and identified impaired nutritional status, defined by scores from the Short Nutritional Assessment Questionnaire (SNAQ) or low Body Mass Index (BMI), as a significant risk factor for flares (Odds Ratio (OR) 2.61, 95% Confidence Interval (CI) 1.02–6.69) [30]. Similarly, the authors of a Swedish study found that inadequate energy intake during hospitalization for acute severe flares, particularly in older patients, was independently associated with shorter time to relapse [31].

Beyond general nutritional status, body composition has also emerged as a potential predictor of disease flare. Sehgal et al. demonstrated that a higher Visceral Adipose Tissue–Subcutaneous Adipose Tissue (VAT:SAT) ratio (≥1.0) was strongly associated with shorter time to flare (hazard ratio (HR) 4.8), while BMI itself was not predictive [32]. Expanding on this, Jain et al. reported that obesity was associated with increased odds of persistent disease activity or relapse in both CD and UC [33]. Furthermore, obesity was linked to poorer patient-reported outcomes, including heightened anxiety, fatigue, and social dysfunction, reinforcing its multifaceted impact on IBD management. In contrast, in a small pilot study, Keshteli et al. found that overweight or obese UC patients were significantly less likely to relapse than those with normal body weight (9.1% vs. 66.7%, *p* = 0.02) [24].

#### 3.2.2. Smoking

Smoking is a widely discussed topic in IBD outpatient clinics, with six studies addressing the impact of smoking in this review. In UC, evidence indicates no clear association between smoking and disease relapse [76]. For example, a nationwide study in the United Kingdom (UK) found no significant differences in outcomes—such as corticosteroid use, thiopurine use, hospitalizations, or colectomy rates—between smokers, ex-smokers, and never-smokers [34]. Additionally, despite 33% of smokers quitting within two years of diagnosis, their outcomes were comparable to those who continued smoking. Similarly, Malibary et al. reported no significant association between smoking habits (cigarette, shisha, or vape) and recurrent UC flares [77]. Importantly, smoking does not appear to improve UC outcomes, and cessation does not worsen the disease, reinforcing the importance of counseling UC patients against smoking.

In contrast with UC, smoking has a well-established negative impact in CD. A multicenter prospective cohort study on CD patients found that continuing smokers had a higher relapse rate and were more likely to be hospitalized compared to non-smokers [35]. Smoking was identified as an independent predictor of relapse, with a hazard ratio of 1.58. Patients who quit smoking had relapse rates similar to those of non-smokers, indicating that smoking cessation can significantly reduce the risk of relapse in CD. Similarly, Gomollón et al. identified smoking as a contributing factor to CD activity, though its predictive impact was lower compared to other variables like cumulative flares in the disease course or age [69].

#### 3.2.3. Sleep

Disturbance of sleep is a common symptom in both active and inactive IBD and is discussed in five studies in this review. Reduced sleep quality increases disease progression. In this review, three studies were identified that discuss sleep disturbance as a risk factor for IBD flares. Uemura et al. reported that sleep disturbances measured using the Pittsburgh Sleep Quality Index (PSQI) increase the odds of disease flares by approximately three-fold (OR 3.09, 95% CI 1.47–6.43) after adjusting for confounding factors [36]. Sofia et al. reproduced these findings in CD patients where they found a positive correlation between poor sleep quality (PSQI > 5) and an increased incidence of active disease at both initial (r = 0.256, *p* = 0.014) and follow-up visits (r = 0.47, *p* = 0.002) [37]. Finally, Ananthakrishnan et al. found twice the odds of relapse in CD patients in remission (95% CI: 1.45–2.76) but found no significant effect of sleep disturbances on flares in UC patients [38]. In regards to factors that affect sleep disturbance, IBD patients with reduced sleep performance reported higher use of immuno-modulators [36]. Moreover, depression, disease activity, smoking, and use of corticosteroids or narcotics, as well as being female, have been identified as significant factors that impact sleep quality [38].

### 3.3. Pharmacological Influences

Ten studies explored the impact of medications on IBD relapse, identifying both triggers and potential protective factors. The role of non-steroidal anti-inflammatory drugs (NSAIDs) is complex and context-dependent. In CD, acetic acid derivatives and related substances, a subset of NSAIDs, were identified as potential risk factors for relapse in a predictive model developed by Gomollón et al. (2022) with a sample size of nearly 6000 patients. However, their relative importance in the model was modest [69]. In UC, NSAID use was linked to an increased risk of relapse (HR 6.41, 95% CI 1.88–21.9) [75]. Conversely, Hensley et al. found that Cyclooxygenase-2 (COX-2) inhibitors and non-selective NSAIDs were inversely associated with relapse in UC patients (adjusted OR 0.06, 95% CI 0.01–0.50), though this effect was not observed in CD patients (adjusted OR 1.25, 95% CI 0.18–7.46) [39]. It should be noted that the sample size of this study was relatively small, with only 34 patients using NSAIDs. These findings highlight the variability in the effects of NSAIDs and COX inhibitors on IBD relapse, which may depend on factors such as drug type, dosage, study design, and sample size. This variability underscores the need for further research to clarify these associations.

The use of antibiotics has also been shown to influence IBD relapse rates. Lo et al. demonstrated that specific antibiotics, such as quinolones (OR 3.82) and beta-lactam antibiotics (OR 1.36), significantly increased the risk of flare-ups [40]. However, the indication for prescribing these antibiotics was not taken into account. A study from North India found higher antibiotic use in the past three months among UC patients with a disease flare compared to those in remission (29.4% vs. 6.1%), underscoring the need for cautious antibiotic management [41]. Interestingly, this study also reported a similar incidence of recent infections in both groups, suggesting that the association between antibiotic use and flares may not be attributable to the underlying indication for antibiotics.

Willemze et al. identified β-blocker use as a potential risk factor for IBD relapse, with an increased hazard ratio of 1.54 (95% CI 1.05–2.25) [42]. Additionally, opioid use, as shown by Riggott et al., is linked with adverse outcomes in IBD, including a heightened risk of intestinal resection (HR 7.09), particularly when codeine and dihydrocodeine were excluded (HR 42.9) [43]. Belladonna derivatives, used with analgesics, and proton pump inhibitors, commonly prescribed for gastrointestinal symptoms, were both associated with an increased risk of CD relapse in a small pilot study [69]. These findings emphasize the importance of careful medication management and highlight the need to address comorbidities in IBD care.

Cancer treatments also influence IBD relapse, as demonstrated by Axelrad et al., whose studies showed that hormone therapy increased relapse risk (HR 2.00 for monotherapy; HR 1.86 for combination therapy), whereas cytotoxic chemotherapy helped maintain remission in 75% of patients [44,45]. These findings suggest that the therapeutic regimen for patients with IBD undergoing cancer treatment should be closely coordinated between the IBD and oncologic care providers.

The risk of polypharmacy, where patients use multiple non-IBD medications, further complicates IBD management. Wang et al. found that major polypharmacy (≥5 non-UC medications) significantly increased the risk of disease flare in UC patients (OR 4.00) [46]. This highlights the potential risks of medication burden, especially in older patients or those with comorbidities. However, Wang et al. found no association between polypharmacy and therapy escalation, hospitalization, or surgery.

The use of complementary and alternative medicine (CAM) also appears to be associated with the occurrence of UC flares, as shown in a study from North India, with CAM use being significantly higher in patients with flares (70.6% vs. 33.3%) [41]. This underscores the challenges of managing UC in regions with limited access to conventional treatments, where CAM is often used as an alternative.

Taken together, these studies emphasize the importance of considering both individual medications and polypharmacy when managing IBD. Variability in findings, particularly concerning NSAIDs and COX inhibitors, suggests the need for personalized approaches based on IBD subtype, medication dosage, and patient characteristics. Proton pump inhibitors are often initiated but rarely discontinued, possibly due to the incorrect perception that long-term use is safe. Future research should explore the underlying mechanisms of drug–disease interactions and guide medication management to minimize relapse risk while effectively addressing comorbid conditions.

### 3.4. Psychological Factors

#### 3.4.1. Psychological Health

Psychological health is highlighted as a critical factor affecting IBD disease outcomes. This review identified nine studies addressing personality traits, anxiety, depression, and perceived stress as factors influencing disease flares.

Personality types are considered a relevant factor in predicting flares in both UC and CD patients. According to Jordi et al., a personality assessment based on the NEO Five-Factor Inventory (NEO-FFI) has significant predictive power for disease flares [47]. In this prospective cohort study, adjusted NEO-FFI risk scores demonstrated the impact of tendencies toward extraversion-related activity, self-reproach, and neuroticism on the risk of flares in IBD patients. High NEO-FFI scores are associated with high flare endpoints (aHR = 1.53−1.63, corrected *p*-value = 0.003−0.006). Moreover, Lang et al. reported that patients with higher levels of positive emotions have longer flare-free survival (aHR = 0.39, *p* < 0.05) [48].

While some studies indicate that depression is associated with, or may even predict, flares during follow-up in both UC and CD [49,50], other studies report a less consistent relationship [51]. Kochar et al. found an increased risk for disease relapse in depressed patients with CD (relative risk (RR) = 2.3, 95% CI: 1.9–2.8) and UC (RR = 1.3, 95% CI: 0.9–1.7). They emphasize the use of comprehensive assessments, such as the Patient Health Questionnaire-8 (PHQ-8), to identify patients at greater risk of aggressive disease. Similarly, an association between depressive symptoms and recurrent flares was found in a retrospective study conducted in Saudi Arabia [77]. In contrast, Fairbrass et al. found no association between high anxiety or depression and an increased risk of future adverse disease outcomes; however, they observed that individuals with abnormal anxiety and depression had higher healthcare utilization, were more likely to undergo investigations for suspected IBD activity, and were more likely to have elevated clinical disease activity scores.

Three studies explore the impact of perceived stress and social support on disease flares. Sauk et al. reported 3.6 times higher odds of clinical flares (Simple Clinical Colitis Activity Index (SCCAI) > 5) in UC patients with high perceived stress [52]; similarly, Jaghult et al. found that patients experiencing “quite a lot” of stress have a significantly increased risk of acute flares in the near future, with a five-times increased risk compared to days without high stress [53]. Finally, psychological distress patterns such as anxiety (β = 1.443, *p* = 0.008) and low perceived social support (β = 0.903, *p* = 0.0013) are associated with active disease in CD patients. Women reported higher rates of anxiety and somatization than men, highlighting the need for tailored interventions in addressing psychological distress in female IBD patients [54].

Gracie et al. emphasized the bidirectional effect of IBD disease flares and psychological disorders [55]. This is evidenced by abnormal baseline scores for anxiety associated with increased flares and the need for corticosteroid therapy (HR, 2.08; 95% CI, 1.3–3.30) and inversely, an increased risk of anxiety associated with baseline disease activity in both UC and CD (HR = 5.77; 95% CI: 1.89–17.7).

Conclusively, these studies underscore poor psychological health as a predictor for active disease in IBD. Screening for the presence of psychological problems in the outpatient clinic, and the availability of psychological support, may therefore improve both patient well-being and disease course.

#### 3.4.2. Quality of Life

Psychological factors such as stress, anxiety, and depression are linked to quality of life (QoL) in individuals with IBD and are proposed contributors to disease flares. Although no studies examined the direct effect of QoL on disease activity, three articles highlight an association between QoL and an increased risk of flares or more complex disease courses, underscoring the bidirectional relationship between QoL and disease activity. Notably, Li et al. identified key factors influencing QoL, including disease activity, extraintestinal manifestations, and socioeconomic conditions, and noted that lower QoL, occupational stress, and income were predictors of recurrence [56]. Similarly, a New Zealand cohort study found that higher Disease Severity Index scores—based on clinical, biochemical, endoscopic, and treatment-related factors—were associated with impaired QoL, moderate-to-severe psychological distress, and a higher risk of flares within the following year [70]. These findings highlight the critical interplay between psychological and medical factors in IBD management.

Complementary findings are reported in a pilot randomized controlled trial that compares treatment as usual to a project management approach. In the latter approach, patients are encouraged to take a more active role in disease management, including symptom monitoring, medication use, time management, nutrition, and relapse prevention. This intervention improved CD outcomes [57]. They emphasized that effective self-management strategies can enhance QoL by addressing health behaviors that undermine treatment efficacy and increase the risk of disease flares.

### 3.5. COVID-19

#### 3.5.1. Natural Infection

The impact of SARS-CoV-2 infection and vaccination on disease flares is discussed in five studies, with most research indicating no significant effect on flare rates.

Both Neri et al. and Wetwittayakhlang et al. found that SARS-CoV-2 infection did not increase the risk of IBD flare-ups [58,59]. Neri et al. observed no difference in relapse rates (27.5% vs. 21%) between IBD patients with and without recent (<2 months) infection. Similarly, Wetwittayakhlang et al. studied 3516 IBD patients and found that 9.8% of those who contracted COVID-19 experienced an IBD flare within three months. They did not identify specific risk factors for post-COVID flare-ups, reinforcing the notion that SARS-CoV-2 infection itself does not significantly exacerbate IBD disease activity.

#### 3.5.2. Vaccination

When investigating the effect of vaccination, similar results were found. Markovinović et al. and Rossier et al. both reported no increase in flare rates following vaccination [60,61]. Markovinović et al. found no flares within 30 days post-vaccination, and Rossier et al. observed similar flare rates between vaccinated and unvaccinated patients. These results were further confirmed by a large UK cohort (*n* = 1911) in which the authors found that COVID-19 vaccination did not increase flare risk. The adjusted incidence rate ratio for flare was 0.89 (95% CI: 0.77–1.04) over a 3-week follow-up period, indicating no significant association between vaccination and disease flares. [62]. Interestingly, they discovered that for patients with UC, the rate of flare was significantly reduced during the 3 weeks after vaccination.

### 3.6. Environmental Factors

The impact of altitude, climate, and seasonal patterns on IBD flares is analyzed in four retrospective studies and one prospective observational cohort study. More specifically, heat waves, cold spells, and exposure to an altitude of 2000 m above sea level are respectively discussed. In one study performed within the Swiss population, heat waves were found to significantly increase the risk of disease flares in IBD patients (OR = 4.6, 95% CI: 1.6–7.4) [63]. Manser et al. explain this disease mechanism through the expansion of pathogenic bacteria, which is common in hot climates. In contrast, cold spells, defined as a maximum temperature below 0 °C, had no impact on the risk of IBD flares (RR 0.99, 95% CI: 0.72–1.33) [64].

Researchers in Shanghai observed a seasonal pattern for CD, with onset (*p* = 0.015) and relapse (*p* = 0.004) peaking in the months of July and August [65]. However, no such effect was observed for UC. These researchers developed a model that can be useful for predicting IBD relapses based on meteorological data (Mean Square Error = 0.009; Mean Absolute Percentage Error = 17.1%).

In the context of altitude variance, Vavricka et al. reported increased odds of IBD flares within four weeks after patients were exposed to high-altitude regions [66]. This included journeys by flight and relocation. Furthermore, in a prospective study evaluating risk factors for flare-ups during air travel, Park J. et al. found an increased likelihood of experiencing a flare during flight travel in patients with IBD and, in particular, patients with a history of more frequent emergency room visits [67].

### 3.7. Multifactorial Assessments

#### 3.7.1. Clinical Indices

In this review, one study was identified that assessed the use of clinical indices for predicting future flares in UC [68]. The authors studied the relationship between the Endoscopic Clinical Correlation Index (ECCI), Partial Mayo Score (score without endoscopy), SCCAI, and Seo’s Activity Index (SEO) at baseline and the occurrence of a disease flare during a one-year follow-up. Only patients with a severe flare of disease at baseline were excluded from the analysis. Higher index scores were consistently associated with an elevated risk of flares: SEO Activity index (OR = 2.5, 95% CI: 0.85–7.31), SCCAI (OR = 1.71, 95% CI: 0.61–4.85), Partial Mayo Score (OR = 2.0 95% CI: 0.64–6.3), and ECCI (OR = 3.05, 95% CI: 1.14–8.19). However, it should be noted that the study had a small sample size, and flares were defined broadly, including minor symptom exacerbations and therapy adjustments.

#### 3.7.2. Prediction Models

In this review, six studies developing predictive models or risk scoring were identified. These models combine clinical, demographic, psychological, and biochemical factors into algorithms or scoring systems, providing clinicians with tools to predict disease outcomes and tailor treatments accordingly.

Hosseini et al. developed a scoring formula for predicting relapse in UC patients [71]. Key predictors included age at diagnosis, disease activity, previous relapses, and fecal calprotectin levels. A score of ≥6.5 identified patients at elevated risk of relapse, with high sensitivity (80%) and specificity (97.1%). Similarly, Stallmash et al. developed a risk model for CD patients, identifying age at diagnosis (<40 years), anemia, and corticosteroid use at first flare as key predictors of disease complications [72]. This model demonstrated good predictive accuracy, with a sensitivity of 77.7%, specificity of 100%, and overall accuracy of 87.3%.

Cristi Riggot et al. adopted a different approach by using latent class analysis to cluster IBD patients based on both gastrointestinal symptoms (measured using the Harvey–Bradshaw Index (HBI) and SCCAI and psychological symptoms (measured using the Hospital Anxiety and Depression Scale (HADS)) [73]. They identified three clusters, with the highest risk of flare-ups found in the group exhibiting both severe gastrointestinal and psychological symptoms.

Pallota et al. developed multiple risk scoring systems for predicting treatment needs and surgical interventions in CD patients [74]. These systems considered factors like age at diagnosis, disease behavior, BMI, disease activity, and corticosteroid use to help clinicians decide which patients to escalate to corticosteroids, azathioprine, or anti-TNF-α drugs and to identify patients at risk of surgery.

The use of natural language processing and machine learning has also shown promise in improving predictive accuracy. Gollomón et al. analyzed electronic health records from 5938 CD patients and identified smoking, past relapses, and biomarkers like leukocytes, hemoglobin, and fibrinogen as strong predictors of disease relapse [69]. The models achieved over 80% accuracy, although false positives and negatives remained a challenge.

Lastly, Swaminathan et al. developed a Disease Severity Index (DSI) for IBD and explored its association with psychological distress [70]. The study found that higher DSI scores were linked to stress, anxiety, depression, and a more complicated disease course, including the need for more aggressive treatments and surgeries.

## 4. Discussion

While treatment strategies focus on achieving remission, identifying modifiable factors—such as lifestyle choices, psychological health, and environmental exposures—can offer patients additional tools to take control of their disease. In this review, several themes emerged, with nutrition, psychological factors, and non-IBD medication being the most prominent in our findings. While some risk factors were consistently identified across studies, others presented contradictory findings, illustrating the need for further research to elucidate these relationships.

Nutrition is a key modifiable factor in the management of IBD. This review suggests that foods such as red meat, dairy, and processed products may exacerbate symptoms, while fruits, legumes, and poultry could have protective effects. However, findings remain inconsistent, and the 2020 European Society for Clinical Nutrition and Metabolism (ESPEN) guidelines emphasize that no specific dietary restrictions are necessary during remission due to limited evidence [78]. Nevertheless, individual food intolerances are frequently reported by IBD patients, with common triggers including lactose, dairy products, spices, herbs, fried foods, gas-generating foods, and fiber-rich items [79]. Additionally, nutrigenetics may explain why responses to certain nutrients vary between individuals. For instance, different types of fats may interact with cytokine genotypes, influencing disease activity [80]. The relationship between fiber and the microbiome further highlights the complexity of nutritional strategies. While dietary fibers generally promote gut health by fermenting into short-chain fatty acids, patients with active IBD and reduced abundance of fiber-fermenting microbes may experience proinflammatory responses to unfermented fibers [81]. Therefore, a personalized, dietitian-supported approach is crucial for optimizing IBD management. A minimally processed, balanced diet characterized by moderation can serve as a broadly applicable recommendation [82,83].

Other lifestyle factors can also play a role in managing IBD. Smoking increases relapse rates in CD, and cessation significantly reduces the risk of disease flares. However, there is contradictory information on the effects of smoking in UC patients. Despite this uncertainty, widely used IBD guidelines recommend smoking cessation for all patients, given the general health risks [84,85]. While the impact of physical activity, stress management, and psychological support on IBD outcomes and overall well-being has been investigated, these factors are not always fully integrated into current management strategies and guidelines [86].

Beyond modifiable lifestyle factors, environmental exposures also play a role in disease activity. Factors such as heat waves increase flare risks, possibly due to microbiota dysbiosis, while high-altitude exposure and seasonal patterns also influence disease activity, particularly in CD, where summer months see higher relapse rates.

Non-IBD medications like opioids, NSAIDs, antibiotics, and β-blockers and polypharmacy can increase flare risks, raising the likelihood of complications, hospitalizations, and mortality. However, interpreting these risks requires nuance: opioid withdrawal symptoms can mimic IBD flare-ups, complicating clinical assessment. Additionally, studies linking antibiotic use to IBD flares often do not properly consider the underlying indications for the antibiotics [85,87]. The increased risk of a disease flare in the case of polypharmacy warrants attention. Although in this review, no association between polypharmacy and therapy escalation, hospitalization, or surgery was found in a relatively small sample size, it could be argued that polypharmacy might lead healthcare providers to hesitate in escalating therapy. Notably, prior studies have shown that elderly IBD patients are less likely to receive therapy escalation, possibly due to concerns about drug interactions and adverse events [88,89]. Interestingly, COVID-19 infection and vaccination have not been shown to increase flare rates, with one article in this review even suggesting that vaccination may reduce flares in UC. Still, the long-term effects of both infection and vaccination remain uncertain, emphasizing the need for ongoing research to understand their potential impact over time.

Additionally, QoL and psychological well-being emerge as key themes in this review. Baseline anxiety and depression predict flares, therapy escalation, and shorter remission periods. Poor sleep independently raises relapse risk in CD and correlates with lower QoL. The bidirectional nature of QoL and disease flares is evident, as a low QoL can exacerbate IBD symptoms, and, conversely, a disease flare can lead to a decrease in QoL. This emphasizes the importance for clinicians to take tailored treatment approaches that assess and promote overall wellbeing for patients with IBD. This involves integrating comprehensive assessments for the aforementioned factors to effectively monitor patients at risk of flares. These findings align with recommendations from the European Crohn’s and Colitis Organization (ECCO) to use tools for assessing psychological distress and quality of life in IBD [90]. Furthermore, self-management strategies that incorporate personality-driven perspectives, promote better sleep, and address other factors influencing overall well-being can contribute to improved QoL and potentially influence the disease course [57].

Predictive models have become increasingly prominent in the recent literature as a tool to address these challenges. These models integrate a wide range of predictors, including clinical, lifestyle, and environmental factors, to improve the understanding of disease flares. Despite their potential, large interpersonal variation among patients remains a challenge, emphasizing the need for personalization in model application and refinement [91,92].

A potential limitation of this review is the lack of specificity in defining risk categories during the initial search, which may have led to a broad overview rather than a more focused and detailed analysis of each individual topic. However, this broader approach provides a comprehensive perspective, offering practical guidance for clinical consultations. Additionally, it should be noted that the list of factors identified in this review may not be exhaustive, as only one database was used in the initial search. For example, emerging research suggests bidirectional relationships between IBD and other conditions, which were not included in this review. This includes, for instance, the link between periodontitis and apical periodontitis, which may increase the risk of IBD, or the association between comorbid diabetes and worse disease outcomes, including infections, in IBD patients [93,94,95]. Understanding these and other interactions may improve IBD management strategies. Another limitation is the potential for publication bias, as negative or non-significant associations may have been omitted in the included studies. Moreover, the bias assessment revealed methodological inconsistencies across studies, such as variability in flare definitions, sample sizes, and adjustment for confounders. These factors may limit the generalizability of the findings and should be carefully considered when translating results into clinical practice.

While notable findings are identified in this review regarding factors such as altitude variance, personality traits, and prediction modeling, the inconsistency found in several themes and the limited information on the mechanisms suggest the need for more focused, longitudinal research to better understand the mechanisms driving these associations.

## 5. Conclusions

In conclusion, this review provides valuable guidance for clinicians in offering practical advice to IBD patients, helping them take control of modifiable factors that may influence disease management. Personalized treatment strategies that incorporate psychological, environmental, and dietary factors, alongside careful medication management, are essential for optimizing IBD care. Clinicians are encouraged to proactively address mental health by screening for anxiety, depression, and stress while fostering open conversations about lifestyle factors such as smoking and sleep quality. Collaborating with dietitians to provide tailored dietary recommendations and encouraging patient involvement in treatment decisions through shared decision-making can further enhance patient well-being and disease control. By considering the unique needs and conditions of each patient, clinicians can better navigate the complexities of treatment and offer tailored recommendations that empower patients to actively engage in their care.

## Figures and Tables

**Table 1:** Summary of the included studies with their key results.

Author	Study Design	Population	Intervention	Outcome Measure	Key Results
**Nutrition**					
[20]	Cross-sectional Study	Diagnosis: IBD *n* = 103 (Italy)	N.A.	Disease activity (FCP > 150)	↓ Consumption of legumes and potatoes linked to lower relapse risk ↑ Meat consumption linked to higher relapse risk
[21]	Case–Control Study	Diagnosis: IBD *n* = 50 (Shanghai)	N.A.	Association between dietary components and recurrent IBD	↑ Consumption of chilis, fish, milk, nuts, eggs, and fruit linked to higher relapse risk
[22]	Randomized Controlled Trial	Diagnosis: CD *n*= 213	Low-meat vs. high-meat diet	Time to relapse (sCDAI score or treatment changes)	↑ Pro-inflammatory diet associated with higher gut inflammation and relapse risk
[23]	Prospective Observational Study	Diagnosis: UC *n* = 412 (USA)	N.A.	UC relapse within 12 months	↑ Higher intake of fatty acids associated with increased risk of UC relapse and no significant association with processed meats or alcohol
[24]	Prospective Cohort Study (pilot)	Diagnosis: UC *n* = 20 (USA)	N.A.	Clinical UC relapse (partial Mayo score ≥ 3)	↑ Normal weight increases the risk of relapse vs. overweight/obese ↓ Poultry intake linked to lower risk of relapse
[25]	Parallel-group, Cross-over Study	Diagnosis: UC *n* = 17 (USA)	Low-fat diet (LFD) vs. improved standard American diet	Clinical disease activity, inflammatory markers, and microbiome	↑ Low-fat diet reduced inflammation and dysbiosis
[26]	Prospective Cohort	Diagnosis: IBD *n* = 1619 (USA)	N.A.	Disease flare (CDAI or SCCAI)	↓ High fiber intake associated with reduced CD relapses ~ No link with UC relapses
[27]	Randomized Controlled Trial	Diagnosis: UC *n* = 12 (USA)	Carrageenan vs. placebo	UC relapse (SCCAI ≥ 2, treatment escalation)	↑ Carrageenan may aggravate UC and shorten the time to relapse
[28]	Prospective Cohort	Diagnosis: IBD *n*= 40 (Brazil)	N.A.	Biochemical inflammation (FCP, CRP, and fecal zonulin)	↑ Pro-inflammatory diet associated with higher gut inflammation and relapse risk
[29]	Retrospective Cohort	Diagnosis: IBD *n*= 216 (UK)	N.A.	Adverse clinical outcomes (e.g., surgery and steroids)	↑ Zinc and selenium deficiencies predicted adverse outcomes in CD and UC
[30]	Longitudinal Observational Study	Diagnosis: IBD *n* = 417 (The Netherlands)	N.A.	Disease flares (symptom increase combined with biomarkers or imaging)	↑ Nutritional deficiencies increased the likelihood of disease relapses ↑ Low BMI increased the likelihood of disease relapses
[31]	Retrospective Cohort Study	Diagnosis: IBD *n* = 91 (Sweden)	N.A.	Steroid use, readmissions, and highest calprotectin	↓ Low energy intake associated with shorter time to steroid use and relapse
[32]	Retrospective Cohort Study	Diagnosis: IBD *n* = 200 (USA)	N.A.	Time to flare (treatment escalation)	↑ High SAT associated with shorter time to relapse
[33]	Cohort Study	Diagnosis: IBD (USA)	N.A.	Disease activity (sCDAI, SCCAI)	↑ Obesity linked to worsening disease activity
**Smoking**					
[34]	Cohort Study	Diagnosis: UC *n* = 6754 (UK)	N.A.	Corticosteroid use, thiopurine use, hospitalization, and colectomy	~ Smoking and non-smoking patients had similar outcomes for relapses and treatments. Smoking cessation did not worsen disease
[35]	Multicenter Prospective Cohort Study	Diagnosis: CD *n* = 573 (Spain)	N.A.	Clinical relapse, time to relapse, surgery, hospitalization, and medication use	↑ Smokers had higher relapse rates
**Sleep**					
[36]	Cross-sectional Study	Diagnosis: UC or CD *n*= 136 (Japan)	N.A.	IBD flare ((1) more than a 2-point increase in HBI or pMayo, (2) therapy initiation, escalation, or switch, (4) surgery, or (5) hospitalization)	↑ Sleep disturbances increase relapse risk
[37]	Prospective Cohort Study	Diagnosis: CD (tertiary care) *n*= 92 (Controls: *n* = 82) (USA)	N.A.	Hospitalization or surgery	↑ Lower sleep quality scores correlate with increased incidence of active disease
[38]	Prospective Cohort Study	Diagnosis: IBD*n* = 3173 (USA)	N.A.	Clinical disease activity (CDAI or SCCAI scores)	↑ Lower sleep quality associated with increased risk for relapse in CD ↔ No association found in UC patients
**Pharmacological influences**					
[39]	Case–Control Study	Diagnosis: IBD *n* = 158 (UK)	N.A.	Primary outcome: Association between COX inhibitor use and IBD relapse. Separate analyses for UC and CD.	↑ COX-2 inhibitor use associated with reduced relapse in UC but no association for CD
[40]	Nested Case–Control Study	Diagnosis: IBD *n* = 15,636 (Denmark)	N.A.	IBD flares defined as IBD-related hospitalizations	↑ Antibiotics (quinolones and antimycotics) associated with increased relapse risk
[41]	Prospective Case–Control Study	Diagnosis: UC *n* = 84 (India)	N.A.	Flares: SCCAI scores > 5 and evidence of endoscopic activity. Remission: SCCAI scores < 4 and normal fecal calprotectin.	↑ Antibiotic use associated with increased relapse risk. ↑ CAM use associated with increased relapse risk
[42]	Retrospective Case–Control Study	Diagnosis: IBD *n* = 470 (European population)	N.A.	IBD relapse risk identified through IBD medication prescriptions as a proxy relapse rate reported per 1000 person-years HR	↑ β-blocker use linked to higher relapse risk
[43]	Prospective Longitudinal Follow-up Study	Diagnosis: IBD *n* = 1029 (116 Opioid users) (UK)	N.A.	Occurrence of flare, prescription of corticosteroids, treatment escalation, hospitalization, or intestinal resection during 12 months of follow-up	↑ Opioid use linked to psychological comorbidity and higher risk of intestinal resection
[44]	Retrospective Cohort Study	Diagnosis: IBD *n* = 447 (USA)	N.A.	Clinical or endoscopic + histological disease activity after cancer treatment	↑ Hormone therapy linked to increased risk of relapse
[45]	Retrospective Cohort Study	Diagnosis: IBD *n* = 84 (USA)	N.A.	Clinical or endoscopic + histological disease activity after cancer treatment	↑ Hormone therapy, alone or in combination with cytotoxic chemotherapy, increases the risk of IBD reactivation
[46]	Retrospective Cohort Study	Diagnosis: UC *n* = 265 (USA)	N.A.	Disease flare (defined by attending gastroenterologist’s documentation and changes in treatment), therapy escalation, UC-related hospitalization, and surgery within 5 years	↑ Polypharmacy linked to increased risk of relapse
**Psychological health**					
[47]	Prospective Cohort Study	Diagnosis: IBD *n* = 1154 (Switzerland)		Disease flare (clinical disease activity (CDAI or MTWAI), physician-reported flare, new fistula, stenosis, surgery, systemic steroids, therapy start, escalation or switch, new complication, or new EIM	↑ Higher activity, self-reproach, and negative emotions linked to increased disease activity
[48]	Retrospective Cohort Study	Diagnosis: IBD *n*= 702 (Switzerland)	N.A.	Disease flare (clinical disease activity (CDAI or MTWAI), physician-reported flare, new fistula, stenosis, surgery, systemic steroids, therapy start, escalation or switch, new complication, or new EIM	↑ High positive affect scores (Marburg questionnaire >3.5) associated with longer flare-free survival periods
[49]	Prospective Cohort Study	Diagnosis: IBD *n* = 1154 (Switzerland)	N.A.	Disease flare (clinical disease activity (CDAI or MTWAI), physician-reported flare, new fistula, stenosis, surgery, systemic steroids, therapy start, escalation or switch, new complication, or new EIM	↑ Depressive symptoms are associated with increased relapse risk
[50]	Longitudinal Cohort Study	Diagnosis: IBD *n* = 2789 (CD), *n* = 1516 (UC) (USA)	N.A.	Clinical relapse (HBI or SCCAI)	↑ Baseline depression associated with relapse risk
[51]	Longitudinal Cohort Study	Diagnosis: IBD *n* = 1031 (UK)	N.A.	Healthcare utilization and clinical outcomes (number of flares, corticosteroid prescriptions, therapy escalations, hospitalizations, and intestinal resections)	↑ Persistently abnormal or worsening anxiety or depression scores linked to flare risk and more healthcare utilization
[52]	Prospective Longitudinal Study	Diagnosis: UC *n* = 110 (USA)	N.A.	Clinical flares (SCCAI) and biochemical flares (SCCAI ≥5 with fecal calprotectin ≥250 μg/g)	↑ High perceived stress linked to 3.6× higher odds of clinical flare ↔ No difference in biochemical flares between high- and low-stress groups
[53]	Prospective Case-Crossover Study	Diagnosis: IBD *n* = 60 (Sweden)	N.A.	Relapse occurrence (Truelove and Witt criteria: blood in stools, diarrhea, and abdominal pain)	↑ High perceived stress linked to higher risk of relapse
[54]	Cross-sectional Study	Diagnosis: CD *n* = 162 (China)	N.A.	Clinical relapse (CDAI)	↑ Psychological distress and lower social support were significant predictors of disease activity
[55]	Longitudinal Cohort Study	Diagnosis: IBD *n*= 405 (UK)	N.A.	Disease flare (HBI or SCCAI, corticosteroid prescription, therapy escalation, hospitalization, or surgery	↔ Bidirectional effect; abnormal anxiety associated with increased flares. Baseline disease activity associated with increased risk for anxiety
**Quality of Life**					
[56]	Prospective Cohort Study	Diagnosis: IBD *n* = 191 (China)	N.A.	Clinical disease activity (pMayo or CDAI), therapy change, or escalation	↑ Low income, high stress, and poor quality of life predicted disease flares
[57]	Randomized Controlled Trial	Diagnosis: CD *n* = 28 (USA)	Project Management vs. Treatment as Usual	Quality of life (IBDQ), self-efficacy (IBD-SES), stress (PSQ), and medication adherence (MAS)	↓ Project management improved quality of life, self-efficacy, and reduced stress ↓ Project management has the potential to reduce flare risk
**COVID-19**					
[58]	Prospective Cohort Study	Diagnosis: IBD *n* = 118 (Italy)	N.A.	Frequency of IBD relapse within 12 months (clinical relapse, medication change, hospitalization, or need for surgery)	↔ Recent COVID-19 infection did not increase the risk of relapse
[59]	Retrospective Observational Study	Diagnosis: IBD *n* = 3516 (Canada)	N.A.	COVID outcomes (severe/mild), IBD flares (symptoms, HBI, pMayo, an post-COVID infection biomarkers), prevalence of COVID infection in the IBD population vs. the general population	↔ COVID-19 infection did not increase the risk of relapse
[60]	Prospective Cohort Study	Diagnosis: IBD *n* = 316 (Canada)	N.A.	Adverse events, severe adverse events, injection site reactions, and IBD flare	↔ No association found between COVID-19 vaccination and relapses
[61]	Retrospective Cohort Study	Diagnosis: IBD *n* = 396 (Switzerland)	N.A.	Incidence of IBD flares post-vaccination (1-month post-vaccination and after 1 month) and adverse events (self-reported surveys)	↔ No association found between COVID-19 vaccination and relapses
[62]	Self-Controlled Case Series	Diagnosis: IBD *n* = 1911 (UK)	N.A.	IBD flare (defined by primary care consultation with diagnostic coding for IBD, diarrhea, abdominal pain, or rectal bleeding, and corticosteroid prescription)	↔ No association found between COVID-19 vaccination and relapses
**Altitude, Climate, and Seasons**					
[63]	Retrospective Observational Study	Diagnosis: IBD *n* = 2030 (IBD: 736, Infectious gastroenteritis: 786, and Control: 506) (Switzerland)	N.A.	Hospital admission	↑ Heat waves increase the risk of IBD-related hospital admission
[64]	Retrospective Observational Study	Diagnosis: IBD, *n* = 2030 (IBD: 736, Infectious gastroenteritis: 786, and Control: 506) (Switzerland)	N.A.	Hospital admission	↔ No association found between cold spells and IBD flares
[65]	Retrospective Cohort Study and Model Assessment	Diagnosis: IBD *n* = 901 (Shanghai)	N.A.	Monthly patterns of onset and relapse and predictive accuracy of the model	↑ Relapse peaks in from July to August in CD patients ↔ No seasonal patterns observed for UC
[66]	Retrospective Observational Study	Diagnosis: IBD *n* = 103 (Switzerland)	N.A.	Clinical IBD flare (HBI or the Rachmilewitz Index)	↑ Exposure to high altitudes increases the risk of disease flares
[67]	Prospective Observational Study	Diagnosis: IBD *n* = 38 (Seoul)	N.A.	Worsening symptoms or medication prescribed during air travel, with assessment at the first outpatient visit after travel	↑ Flight travel increases the risk of disease flares in patients with IBD comorbidities ↑ Flight travel increases the risk of disease flares in patients with a history of more frequent ER visits
**Clinical Index**					
[68]	Retrospective Cohort Study	Diagnosis: UC *n*= 75 (Italie)	N.A.	Onset or exacerbation of symptoms (abdominal pain, increased bowel movements, blood in stools, and urgency) requiring prompt medical consultation.	↑Higher scores (ECCI, Seo’s Activity Index, SCCAI, and Partial Mayo Score) are associated with a higher risk of disease flares
**Prediction Models**					
[69]	Retrospective Cohort Study	Diagnosis: CD *n* = 5938 (Spain)	N.A.	Clinical relapse and hospitalization	↑ Cumulative past flare, age, past admissions, proton pump inhibitors, smoking, acetic acid derivatives and related substances, and belladonna and derivatives in combination with analgesics
[70]	Prospective Cohort Study	Diagnosis: IBD *n* = 172 (New Zealand)	N.A.	IBD relapse (medication escalation, hospitalization, and surgery)	↑ High Disease Severity Index (DSI) predicted impaired quality of life, moderate-to-severe psychological distress, and increased flare risk
[71]	Prospective Cohort Study	Diagnosis: UC *n* = 157 (Iran)	N.A.	IBD relapse (based on symptoms like bowel movements, bleeding, abdominal pain, and diarrhea)	↑ Fecal calprotectin levels, age, Seo’s Activity Index, and number of previous relapses predicted relapse. Risk-scoring formula developed to predict relapse risk
[72]	Prospective Observational Study	Diagnosis: CD *n* = 341 (Germany)	N.A.	Complicated disease course (need for immunosuppressants, anti-TNF agents, and hospitalization)	↑ Age at onset <40 years, anemia, and treatment with systemic corticosteroids at first flare predicted a complicated disease course
[73]	Longitudinal Follow-up Study	Diagnosis: IBD *n* = 692 (UK)	N.A.	Disease flare (global assessment or corticosteroid use)	↑ Patients with severe gastrointestinal and psychological symptoms had an increased risk of flare or corticosteroid use
[74]	Prospective Cohort Study	Diagnosis: CD *n* = 160 (Italy)	N.A.	Risk of disease progression and need for intensive treatment (steroids, azathioprine, anti-TNF-α drugs, and surgery); flare risk based on clinical and imaging findings	↑ CD complications, small bowel CD lesion >20 cm, absence of colonic–ileal reflux, age <40 years, structuring behavior, and specific intestinal symptoms predicted higher corticosteroid use predictive of intensifying treatment
**Multiple factors**					
[75]	Prospective Cohort Study	Diagnosis: UC *n* = 79 (India)	N.A.	Clinical relapse (all also demonstrated endoscopic relapse)	↑ NSAID use associated with clinical relapse ↑ Higher vitamin A intake associated with clinical relapse
[76]	Prospective Cohort Study	Diagnosis: IBD *n* = 289 (Bulgaria)	N.A.	Frequency of relapses	↑ Younger age increases relapse risk ↑ Shorter disease duration increases relapse risk ↔ No effect of sex ↔ No effect of smoking
[77]	Retrospective Cohort Study	Diagnosis: UC *n* = 89 (Saudi Arabia)	N.A.	Recurrent UC flares	↑ Family history of UC increases the risk of recurrent relapse ↑ Fecal incontinence increases the risk of recurrent relapse ↔ No association with smoking

Abbreviations: IBD, Inflammatory Bowel Disease; UC, Ulcerative Colitis; CD, Crohn’s Disease; N.A., Not Applicable; FCP, Fecal Calprotectin; (s)CDAI, (simplified) Crohn’s Disease Activity Index; SCCAI, Simple Clinical Colitis Activity Index; UK, United Kingdom; USA, United States of America, RCT, Randomized Controlled Trial; COX, Cyclooxygenase; HR, Hazard Ratio; HBI, Harvey–Bradshaw Index; MTWAI, Modified Truelove and Witts Activity Index; EIM, Extra-intestinal Manifestations; PSQ, Perceived Stress Questionnaire; IBDQ, Inflammatory Bowel Disease Questionnaire; IBD-SES, Inflammatory Bowel Disease Self-Efficacy Scale; MAS, Medication Adherence Scale; COVID-19, Coronavirus Disease 2019; CAM, Complementary and Alternative Medicine; DSI, Disease Severity Index; ECCI, Endoscopic Clinical Correlation Index; TNF, Tumor Necrosis Factor; NSAID, Non-steroidal Anti-inflammatory Drugs.

## Abbreviations

The following abbreviations are used in this manuscript:

BMI	Body Mass Index
CAM	Complementary and Alternative Medicine
CD	Crohn’s Disease
CI	Confidence Interval
COVID-19	Coronavirus Disease 2019
COX	Cyclooxygenase
DSI	Disease Severity Index
ECCI	Endoscopic Clinical Correlation Index
ECCO	European Crohn’s and Colitis Organization
ESPEN	European Society for Clinical Nutrition and Metabolism
HR	Hazard Ratio
IBD	Inflammatory Bowel Disease
MeSH	Medical Subject Headings
MMAT	Mixed Methods Appraisal Tool
NEO-FFI	NEO Five-Factor Inventory
NSAID	Non-steroidal Anti-inflammatory Drug
OR	Odds Ratio
PHQ-8	Patient Health Questionnaire-8
PSQI	Pittsburgh Sleep Quality Index
QoL	Quality of Life
RCT	Randomized Controlled Trial
RR	Relative Risk
SCCAI	Simple Clinical Colitis Activity Index
SEO	Seo’s Activity Index
SNAQ	Short Nutritional Assessment Questionnaire
TNF	Tumor Necrosis Factor
UC	Ulcerative Colitis
UK	United Kingdom
USA	United States of America
VAT:SAT	Visceral Adipose Tissue–Subcutaneous Adipose Tissue

## Appendix A. Original Search Terms

“Inflammatory Bowel Diseases” [Mesh] OR “Inflammatory Bowel Disease” [tiab] OR “Idiopathic Proctocolitis” [tiab] OR “Ulcerative Colitis” [tiab] OR “Colitis Gravis” [tiab] OR “Crohn’s Enteritis” [tiab] OR “Regional Enteritis” [tiab] OR “Crohn’s Disease” [tiab] OR “Crohn’s Disease” [tiab] OR “Ileocolitis” [tiab] OR “Granulomatous Colitis” [tiab] OR “Terminal Ileitis” [tiab] OR “Regional Ileitis” [tiab] OR “Colitis” [tiab]

AND

“Recurrence” [Mesh] OR “Recurrences” [tiab] OR “Recrudescence” [tiab] OR “Recrudescence” [tiab] OR “Relapse” [tiab] OR “Relapses” [tiab] OR “Flare” [tiab] OR “Flares” [tiab] OR “Flare up” [tiab] OR “Symptom exacerbation*” [tiab] OR “Flareup” [tiab] OR “Flare-up” [tiab]

AND

“Risk Factors” [Mesh] OR “Risk Factors” [tiab] OR “Risk” [tiab] OR “Predictive Value of Tests” [Mesh] OR “Predictive Value of Tests” [tiab] OR “Predict*” [tiab]

## Appendix B. Mixed Methods Appraisal Tool Quality Assessment

**Table A1:** Quantitative randomized controlled trial quality assessment.

Study Title	S1. Are There Clear Research Questions?	S2. Do the Collected Data Allow Us to Address the Research Questions?	2.1. Is Randomization Appropriately Performed?	2.2. Are the Groups Comparable at Baseline?	2.3. Are There Complete Outcome Data?	2.4. Are Outcome Assessors Blinded to the Intervention Provided?	2.5 Did the Participants Adhere to the Assigned Intervention?
A diet low in red and processed meat does not reduce rate of Crohn’s disease flares [22]	+	+	+	−	+	−	−
A randomized trial of the effects of the no-carrageenan diet on ulcerative colitis disease activity [27]	+	+	+	+	+	+	+
Optimizing management of Crohn’s disease within a project management framework: results of a pilot study [57]	+	+	+/−	+	+/−	−	+

**Table A2:** Quantitative non-randomized trial quality assessment.

Study	S1. Are There Clear Research Questions?	S2. Do the Collected Data Allow Us to Address the Research Questions?	3.1. Are the Participants Representative of the Target Population?	3.2. Are Measurements Appropriate Regarding Both the Outcome and Intervention (or Exposure)?	3.3. Are There Complete Outcome Data?	3.4. Are the Confounders Accounted for in the Design and Analysis?	3.5. During the Study Period, is the Intervention Administered (or Exposure Occurred) as Intended?
Influence of diet on the course of inflammatory bowel disease [20]	+	+	−	+	+	+/−	+
Dietary risk factors for inflammatory bowel disease in Shanghai: A case-control study [21]	+	+	+	+	+	+	+
High dietary intake of specific fatty acids increases risk of flares in patients with ulcerative colitis in remission during treatment with aminosalicylates [23]	+	+	+	+	+	+	+
Dietary and metabolomic determinants of relapse in ulcerative colitis patients: a pilot prospective cohort study [24]	+	+	−	+	+	+	+
Low-fat, high-fiber diet reduces markers of inflammation and dysbiosis and improves quality of life in patients with ulcerative colitis [25]	+	+	−	+	+	+	+
Avoidance of fiber is associated with greater risk of Crohn’s disease flare in a 6-month period [26]	+	+	+	+	+	−	+
Pro-inflammatory diet is correlated with high veillonella rogosae, gut inflammation and clinical relapse of inflammatory bowel disease [28]	+	+	+	+	+	+	+
Micronutrient status and prediction of disease outcome in adults with inflammatory bowel disease receiving biologic therapy [29]	+	+	+	+	+	+	+
Risk of impaired nutritional status and flare occurrence in IBD outpatients [30]	+	+	+	+	+	−	+
Older age is a risk factor for inadequate energy intake during acute, severe IBD and is associated with shorter time to relapse [31]	+	+	+/−	+	+/−	+	+
Visceral adiposity independently predicts time to flare in inflammatory bowel disease but body mass index does not [32]	+	+	+	+	+	+	+
Impact of obesity on disease activity and patient-reported outcomes measurement information system (PROMIS) in inflammatory bowel diseases [33]	+	+	+	+/−	+	+	+
The impact of smoking and smoking cessation on disease outcomes in ulcerative colitis: a nationwide population-based study [34]	+	+	+	+	+	+	+
Impact of smoking cessation on the clinical course of Crohn’s disease under current therapeutic algorithms: a multicenter prospective study [35]	+	+	+	+	+	+	+
Poor sleep quality in Crohn’s disease is associated with disease activity and risk for hospitalization or surgery [37]	+	+	−	+	+	+	+
Sleep disturbance and risk of active disease in patients with Crohn’s disease and ulcerative colitis [38]	+	+	+	+	+	+	+
Use of cyclo-oxygenase inhibitors is not associated with clinical relapse in inflammatory bowel disease: a case-control study [39]	+	+	+	+	+	+	+
Specific Antibiotics Increase the Risk of Flare-Ups in Patients with Inflammatory Bowel Disease: Results from a Danish Nationwide Population-Based Nested Case-Control Study [40]	+	+	+	+	+	+	+
Factors contributing to flares of ulcerative colitis in North India-a case-control study [41]	+	+	+	+	+	+	+
β-Blocker use is associated with a higher relapse risk of inflammatory bowel disease: a Dutch retrospective case–control study [42]	+	+	+	+/−	+	+/−	+
Impact of Opioid Use on the Natural History of Inflammatory Bowel Disease: Prospective Longitudinal Follow-up Study [43]	+	+	+	+	+	+	+
Hormone therapy for cancer is a risk factor for relapse of inflammatory bowel diseases [44]	+	+	+	+	+	+	+
Effects of cancer treatment on inflammatory bowel disease remission and reactivation [45]	+	+	+/−	+	+	+	+
Polypharmacy is a risk factor for disease flare in adult patients with ulcerative colitis: a retrospective cohort study [46]	+	+	+/−	+	+	+	+
The personality traits activity, self-reproach, and negative affect jointly predict clinical recurrence, depressive symptoms, and low quality of life in inflammatory bowel disease patients [47]	+	+	+	+	+	+	+
Because I’m happy–positive affect and its predictive value for future disease activity in patients with inflammatory bowel diseases: a retrospective cohort study [48]	+	+	+	+	+	+	+
Depressive symptoms predict clinical recurrence of inflammatory bowel disease [49]	+	+	+	+	+	+	+
Depression is associated with more aggressive inflammatory bowel disease [50]	+	+	+	+	+	+	+
Characteristics and effect of anxiety and depression trajectories in inflammatory bowel disease [51]	+	+	+	+	+	+	+
High perceived stress is associated with increased risk of ulcerative colitis clinical flares [52]	+	+	+	+	+	+	+
Stress as a trigger for relapses in IBD: a case-crossover study [53]	+	+	+/−	+	+	+	+
Is disease activity associated with social support and psychological distress in Crohn’s disease patients? Results of a cross-sectional study in a Chinese hospital population [54]	+	+	−	−	+/−	+	+
Bi-directionality of brain–gut interactions in patients with inflammatory bowel disease [55]	+	+	+	+	+	+	+
Constructing a prediction model of inflammatory bowel disease recurrence based on factors affecting the quality of life [56]	+	+	−	+	+	+	+
Severe acute respiratory syndrome coronavirus 2 infection does not worsen the course of inflammatory bowel disease in the long term [58]	+	+	+	+	+	+	+
Clinical Outcomes of COVID-19 and Impact on Disease Course in Patients with Inflammatory Bowel Disease [59]	+	+	+	+	+	+	+
SARS-CoV-2 vaccination in inflammatory bowel disease patients is not associated with flares: a retrospective single-centre Swiss study [61]	+	+	+/−	+	+	+	+
Heat waves, incidence of infectious gastroenteritis, and relapse rates of inflammatory bowel disease: a retrospective controlled observational study [63]	+	+	+	+	+	+	+
The impact of cold spells on the incidence of infectious gastroenteritis and relapse rates of inflammatory bowel disease: a retrospective controlled observational study [64]	+	+	+/−	+/−	+	+	+/−
Seasonal variation in onset and relapse of IBD and a model to predict the frequency of onset, relapse, and severity of IBD based on artificial neural network [65]	+	+	+/−	+	−	−	+
High altitude journeys and flights are associated with an increased risk of flares in inflammatory bowel disease patients [66]	+	+	+	+	+	+	+
Clinical factors to predict flare-up in patients with inflammatory bowel disease during international air travel: A prospective study [67]	+	+	+/−	+	+	+	+
Application of clinical indexes in ulcerative colitis patients in regular follow-up visit. correlation with endoscopic ‘mucosal healing’ and implication for management [68]	+	+	+/−	+/−	+	−	+
Clinical characteristics and prognostic factors for Crohn’s disease relapses using natural language processing and machine learning: a pilot study [69]	+	+	+	+	+/−	+/−	+
The disease severity index for inflammatory bowel disease is associated with psychological symptoms and quality of life, and predicts a more complicated disease course [70]	+	+	+	+	+	+	+
Developing a novel risk-scoring system for predicting relapse in patients with ulcerative colitis: A prospective cohort study [71]	+	+	+	+	+	+	+
Predictive parameters for the clinical course of Crohn’s disease: development of a simple and reliable risk model [72]	+	+	−	+	+	+	+
Novel symptom clusters predict disease impact and healthcare utilisation in inflammatory bowel disease: Prospective longitudinal follow-up study [73]	+	+	+	+	+	+	+
A risk score system to timely manage treatment in Crohn’s disease: a cohort study [74]	+	+	+/−	+	+	+	+
Evaluating clinical, dietary, and psychological risk factors for relapse of ulcerative colitis in clinical, endoscopic, and histological remission [75]	+	+	−	+	+	+	+
Young age and short duration of the disease are associated with more frequent relapses in inflammatory bowel disease patients [76]	+	+	+	+	+/−	−	+
Factors affecting ulcerative colitis flare-ups: associations with smoking habits and other patient characteristics [77]	+	+	+	+	+	+	+

**Table A3:** Quantitative descriptive trial quality assessment.

Study	S1. Are There Clear Research Questions?	S2. Do the Collected Data Allow Us to Address the Research Questions?	4.1. Is the Sampling Strategy Relevant to Address the Research Question?	4.2. Is the Sample Representative of the Target Population?	4.3. Are the Measurements Appropriate?	4.4. Is the Risk of Non-response Bias Low?	4.5. Is the Statistical Analysis Appropriate to Answer the Research Question?
Sleep disturbances in Japanese patients with inflammatory bowel disease and their impact on disease flare [36]	+	+	+	+	+	+/−	+
Adverse events and serological responses after SARS-CoV-2 vaccination in individuals with inflammatory bowel disease [60]	+	+	+	+	+	−	+
Is vaccination against COVID-19 associated with inflammatory bowel disease flare? self-controlled case series analysis using the UK CPRD [62]	+	+	+	+	+	+	+

## References

[1] Rubin D.T., Torres J., Dotan I., Xu L.T., Modesto I., Woolcott J.C., Gardiner S., Sands B.E. (2024). An insight into patients’ perspectives of ulcerative colitis flares via analysis of online public forum posts. Inflamm. Bowel Dis..

[2] Al Khoury A., Balram B., Bessissow T., Afif W., Gonczi L., Abreu M., Lakatos P.L. (2022). Patient perspectives and expectations in inflammatory bowel disease: A systematic review. Dig. Dis. Sci..

[3] Abraham C., Cho J.H. (2009). Inflammatory bowel disease. New Engl. J. Med..

[4] Singh N., Bernstein C.N. (2022). Environmental risk factors for inflammatory bowel disease. United Eur. Gastroenterol. J..

[5] Ananthakrishnan A.N. (2015). Epidemiology and risk factors for IBD. Nat. Rev. Gastroenterol. Hepatol..

[6] Martin T.D., Chan S.S., Hart A.R. (2015). Environmental factors in the relapse and recurrence of inflammatory bowel disease: A review of the literature. Dig. Dis. Sci..

[7] Black J., Sweeney L., Yuan Y., Singh H., Norton C., Czuber-Dochan W. (2022). Systematic review: The role of psychological stress in inflammatory bowel disease. Aliment. Pharmacol. Ther..

[8] De Sousa J.F.M., Paghdar S., Khan T.M., Patel N.P., Chandrasekaran S., Tsouklidis N., Paghdar S., Khan T.M. (2022). Stress and inflammatory bowel disease: Clear mind, happy colon. Cureus.

[9] Peters V., Spooren C.E., Pierik M.J., Weersma R.K., van Dullemen H.M., Festen E.A., Visschedijk M.C., Masclee A.A., Hendrix E.M., Almeida R.J. (2021). Dietary intake pattern is associated with occurrence of flares in IBD patients. J. Crohn’s Colitis.

[10] Torres J., Bonovas S., Doherty G., Kucharzik T., Gisbert J.P., Raine T., Adamina M., Armuzzi A., Bachmann O., Bager P. (2020). ECCO guidelines on therapeutics in Crohn’s disease: Medical treatment. J. Crohn’s Colitis.

[11] Raine T., Bonovas S., Burisch J., Kucharzik T., Adamina M., Annese V., Bachmann O., Bettenworth D., Chaparro M., Czuber-Dochan W. (2022). ECCO guidelines on therapeutics in ulcerative colitis: Medical treatment. J. Crohn’s Colitis.

[12] Peyrin–Biroulet L., Bressenot A., Kampman W. (2014). Histologic remission: The ultimate therapeutic goal in ulcerative colitis?. Clin. Gastroenterol. Hepatol..

[13] Sandborn W.J., Hanauer S., Van Assche G., Panés J., Wilson S., Petersson J., Panaccione R. (2014). Treating beyond symptoms with a view to improving patient outcomes in inflammatory bowel diseases. J. Crohn’s Colitis.

[14] Pariente B., Cosnes J., Danese S., Sandborn W.J., Lewin M., Fletcher J.G., Chowers Y., d’Haens G., Feagan B.G., Hibi T. (2011). Development of the Crohn’s disease digestive damage score, the Lemann score. Inflamm. Bowel Dis..

[15] Peyrin-Biroulet L., Cieza A., Sandborn W.J., Coenen M., Chowers Y., Hibi T., Kostanjsek N., Stucki G., Colombel J.-F., the International Programme to Develop New Indexes for Crohn’s Disease (IPNIC) Group (2012). Development of the first disability index for inflammatory bowel disease based on the international classification of functioning, disability and health. Gut.

[16] Torres J., Billioud V., Sachar D.B., Peyrin-Biroulet L., Colombel J.F. (2012). Ulcerative colitis as a progressive disease: The forgotten evidence. Inflamm. Bowel Dis..

[17] Khanna R., Bressler B., Levesque B.G., Zou G., Stitt L.W., Greenberg G.R., Panaccione R., Bitton A., Paré P., Vermeire S. (2015). Early combined immunosuppression for the management of Crohn’s disease (REACT): A cluster randomised controlled trial. Lancet.

[18] Page M.J., McKenzie J.E., Bossuyt P.M., Boutron I., Hoffmann T.C., Mulrow C.D., Shamseer L., Tetzlaff J.M., Akl E.A., Brennan S.E. (2021). The PRISMA 2020 statement: An updated guideline for reporting systematic reviews. BMJ.

[19] Hong Q.N., Fàbregues S., Bartlett G., Boardman F., Cargo M., Dagenais P., Gagnon M.-P., Griffiths F., Nicolau B., O’Cathain A. (2018). The Mixed Methods Appraisal Tool (MMAT) version 2018 for information professionals and researchers. Educ. Inf..

[20] Tasson L., Canova C., Vettorato M.G., Savarino E., Zanotti R. (2017). Influence of diet on the course of inflammatory bowel disease. Dig. Dis. Sci..

[21] Mi L., Zhang C., Yu X.-f., Zou J., Yu Y., Bao Z.-J. (2022). Dietary risk factors for inflammatory bowel disease in Shanghai: A case-control study. Asia Pac. J. Clin. Nutr..

[22] Albenberg L., Brensinger C.M., Wu Q., Gilroy E., Kappelman M.D., Sandler R.S., Lewis J.D. (2019). A diet low in red and processed meat does not reduce rate of Crohn’s disease flares. Gastroenterology.

[23] Barnes E.L., Nestor M., Onyewadume L., de Silva P.S., Korzenik J.R., Aguilar H., Bailen L., Berman A., Bhaskar S.K., Brown M. (2017). High dietary intake of specific fatty acids increases risk of flares in patients with ulcerative colitis in remission during treatment with aminosalicylates. Clin. Gastroenterol. Hepatol..

[24] Keshteli A.H., van den Brand F.F., Madsen K.L., Mandal R., Valcheva R., Kroeker K.I., Han B., Bell R.C., Cole J., Hoevers T. (2017). Dietary and metabolomic determinants of relapse in ulcerative colitis patients: A pilot prospective cohort study. World J. Gastroenterol..

[25] Fritsch J., Garces L., Quintero M.A., Pignac-Kobinger J., Santander A.M., Fernández I., Ban Y.J., Kwon D., Phillips M.C., Knight K. (2021). Low-fat, high-fiber diet reduces markers of inflammation and dysbiosis and improves quality of life in patients with ulcerative colitis. Clin. Gastroenterol. Hepatol..

[26] Brotherton C.S., Martin C.A., Long M.D., Kappelman M.D., Sandler R.S. (2016). Avoidance of fiber is associated with greater risk of Crohn’s disease flare in a 6-month period. Clin. Gastroenterol. Hepatol..

[27] Bhattacharyya S., Shumard T., Xie H., Dodda A., Varady K.A., Feferman L., Halline A.G., Goldstein J.L., Hanauer S.B., Tobacman J.K. (2017). A randomized trial of the effects of the no-carrageenan diet on ulcerative colitis disease activity. Nutr. Healthy Aging.

[28] Rocha I.M.G.d., Torrinhas R., Fonseca D., Lyra C.d.O., de Sousa Alves Neri J.L., Balmant B.D., Callado L., Charlton K., Queiroz N., Waitzberg D.L. (2023). Pro-inflammatory diet is correlated with high veillonella rogosae, gut inflammation and clinical relapse of inflammatory bowel disease. Nutrients.

[29] Brownson E., Saunders J., Jatkowska A., White B., Gerasimidis K., Seenan J.P., Macdonald J. (2024). Micronutrient status and prediction of disease outcome in adults with inflammatory bowel disease receiving biologic therapy. Inflamm. Bowel Dis..

[30] Spooren C.E., Wintjens D.S., de Jong M.J., van der Meulen-de A.E., Romberg-Camps M.J., Becx M.C., Maljaars J.P., van Bodegraven A.A., Mahmmod N., Markus T. (2019). Risk of impaired nutritional status and flare occurrence in IBD outpatients. Dig. Liver Dis..

[31] Kulmala K.A., Björk J., Andersson S., Backman A.-S., Eberhardson M., Bresso F., Hedin C.R. (2020). Older age is a risk factor for inadequate energy intake during acute, severe IBD and is associated with shorter time to relapse. Scand. J. Gastroenterol..

[32] Sehgal P., Su S., Zech J., Nobel Y., Luk L., Economou I., Shen B., Lewis J.D., Freedberg D.E. (2024). Visceral adiposity independently predicts time to flare in inflammatory bowel disease but body mass index does not. Inflamm. Bowel Dis..

[33] Jain A., Nguyen N.H., Proudfoot J.A., Martin C.F., Sandborn W.J., Kappelman M.D., Long M.D., Singh S. (2019). Impact of obesity on disease activity and patient-reported outcomes measurement information system (PROMIS) in inflammatory bowel diseases. Off. J. Am. Coll. Gastroenterol..

[34] Blackwell J., Saxena S., Alexakis C., Bottle A., Cecil E., Majeed A., Pollok R.C. (2019). The impact of smoking and smoking cessation on disease outcomes in ulcerative colitis: A nationwide population-based study. Aliment. Pharmacol. Ther..

[35] Nunes T., Etchevers M.J., García-Sánchez V., Ginard D., Martí E., Barreiro-de Acosta M., Gomollón F., Arroyo M., Bastida G., Gonzalez B. (2016). Impact of smoking cessation on the clinical course of Crohn’s disease under current therapeutic algorithms: A multicenter prospective study. Off. J. Am. Coll. Gastroenterol..

[36] Uemura R., Fujiwara Y., Iwakura N., Shiba M., Watanabe K., Kamata N., Yamagami H., Tanigawa T., Watanabe T., Tominaga K. (2016). Sleep disturbances in Japanese patients with inflammatory bowel disease and their impact on disease flare. SpringerPlus.

[37] Sofia M.A., Lipowska A.M., Zmeter N., Perez E., Kavitt R., Rubin D.T. (2020). Poor sleep quality in Crohn’s disease is associated with disease activity and risk for hospitalization or surgery. Inflamm. Bowel Dis..

[38] Ananthakrishnan A.N., Long M.D., Martin C.F., Sandler R.S., Kappelman M.D. (2013). Sleep disturbance and risk of active disease in patients with Crohn’s disease and ulcerative colitis. Clin. Gastroenterol. Hepatol..

[39] Hensley A., Beales I.L. (2015). Use of cyclo-oxygenase inhibitors is not associated with clinical relapse in inflammatory bowel disease: A case-control study. Pharmaceuticals.

[40] Lo B., Biederman L., Rogler G., Dora B., Kreienbühl A., Vind I., Bendtsen F., Burisch J. (2024). Specific Antibiotics Increase the Risk of Flare-Ups in Patients with Inflammatory Bowel Disease: Results from a Danish Nationwide Population-Based Nested Case-Control Study. J. Crohn’s Colitis.

[41] Rana V.S., Mahajan G., Patil A.N., Singh A.K., Jearth V., Sekar A., Singh H., Saroch A., Dutta U., Sharma V. (2023). Factors contributing to flares of ulcerative colitis in North India-a case-control study. BMC Gastroenterol..

[42] Willemze R.A., Bakker T., Pippias M., Ponsioen C.Y., de Jonge W.J. (2018). β-Blocker use is associated with a higher relapse risk of inflammatory bowel disease: A Dutch retrospective case–control study. Eur. J. Gastroenterol. Hepatol..

[43] Riggott C., Fairbrass K.M., Selinger C.P., Gracie D.J., Ford A.C. (2024). Impact of Opioid Use on the Natural History of Inflammatory Bowel Disease: Prospective Longitudinal Follow-up Study. Inflamm. Bowel Dis..

[44] Axelrad J.E., Bazarbashi A., Zhou J., Castañeda D., Gujral A., Sperling D., Glass J., Agrawal M., Hong S., Lawlor G. (2020). Hormone therapy for cancer is a risk factor for relapse of inflammatory bowel diseases. Clin. Gastroenterol. Hepatol..

[45] Axelrad J.E., Fowler S.A., Friedman S., Ananthakrishnan A.N., Yajnik V. (2012). Effects of cancer treatment on inflammatory bowel disease remission and reactivation. Clin. Gastroenterol. Hepatol..

[46] Wang J., Nakamura T.I., Tuskey A.G., Behm B.W. (2019). Polypharmacy is a risk factor for disease flare in adult patients with ulcerative colitis: A retrospective cohort study. Intest. Res..

[47] Jordi S.B.U., Lang B.M., Wyss J., Auschra B., Yilmaz B., Krupka N., Greuter T., Schreiner P., Biedermann L., Preisig M. (2022). The personality traits activity, self-reproach, and negative affect jointly predict clinical recurrence, depressive symptoms, and low quality of life in inflammatory bowel disease patients. J. Gastroenterol..

[48] Lang B.M., Ledergerber M., Jordi S.B.U., Krupka N., Biedermann L., Schreiner P., Juillerat P., Wyss J., Vavricka S.R., Zeitz J. (2023). Because I’m happy–positive affect and its predictive value for future disease activity in patients with inflammatory bowel diseases: A retrospective cohort study. Ther. Adv. Gastroenterol..

[49] Jordi S.B.U., Lang B.M., Auschra B., von Känel R., Biedermann L., Greuter T., Schreiner P., Rogler G., Krupka N., Sulz M.C. (2022). Depressive symptoms predict clinical recurrence of inflammatory bowel disease. Inflamm. Bowel Dis..

[50] Kochar B., Barnes E.L., Long M.D., Cushing K.C., Galanko J., Martin C.F., Raffals L.E., Sandler R.S. (2018). Depression is associated with more aggressive inflammatory bowel disease. Off. J. Am. Coll. Gastroenterol..

[51] Fairbrass K.M., Guthrie E.A., Black C.J., Selinger C.P., Gracie D.J., Ford A.C. (2023). Characteristics and effect of anxiety and depression trajectories in inflammatory bowel disease. Off. J. Am. Coll. Gastroenterol..

[52] Sauk J.S., Ryu H.J., Labus J.S., Khandadash A., Ahdoot A.I., Lagishetty V., Katzka W., Wang H., Naliboff B., Jacobs J.P. (2023). High perceived stress is associated with increased risk of ulcerative colitis clinical flares. Clin. Gastroenterol. Hepatol..

[53] Jaghult S., Saboonchi F., Moller J., Johansson U.-B., Wredling R., Kapraali M. (2013). Stress as a trigger for relapses in IBD: A case-crossover study. Gastroenterol. Res..

[54] Huang M., Tu L., Wu L., Zou Y., Li X., Yue X., Huang C., Lei P., Li Q., Han P. (2023). Is disease activity associated with social support and psychological distress in Crohn’s disease patients? Results of a cross-sectional study in a Chinese hospital population. BMJ Open.

[55] Gracie D.J., Guthrie E.A., Hamlin P.J., Ford A.C. (2018). Bi-directionality of brain–gut interactions in patients with inflammatory bowel disease. Gastroenterology.

[56] Li M., Tao Y., Sun Y., Wu J., Zhang F., Wen Y., Gong M., Yan J., Liang H., Bai X. (2023). Constructing a prediction model of inflammatory bowel disease recurrence based on factors affecting the quality of life. Front. Med..

[57] Keefer L., Doerfler B., Artz C. (2012). Optimizing management of Crohn’s disease within a project management framework: Results of a pilot study. Inflamm. Bowel Dis..

[58] Neri B., D’Agostini G., Salvatori S., Mossa M., Bettin F., Mancone R., Marafini I., Lolli E., Calabrese E., Monteleone G. (2023). Severe acute respiratory syndrome coronavirus 2 infection does not worsen the course of inflammatory bowel disease in the long term. Eur. J. Gastroenterol. Hepatol..

[59] Wetwittayakhlang P., Albader F., Golovics P.A., Hahn G.D., Bessissow T., Bitton A., Afif W., Wild G., Lakatos P.L. (2021). Clinical Outcomes of COVID-19 and Impact on Disease Course in Patients with Inflammatory Bowel Disease. Can. J. Gastroenterol. Hepatol..

[60] Markovinović A., Quan J., Herauf M., Hracs L., Windsor J.W., Sharifi N., Coward S., Caplan L., Gorospe J., Ernest-Suarez K. (2023). Adverse events and serological responses after SARS-CoV-2 vaccination in individuals with inflammatory bowel disease. Off. J. Am. Coll. Gastroenterol..

[61] Rossier L.N., Décosterd N.P., Matter C.B., Staudenmann D.A., Moser A., Egger B., Seibold F.W. (2024). SARS-CoV-2 vaccination in inflammatory bowel disease patients is not associated with flares: A retrospective single-centre Swiss study. Ann. Med..

[62] Card T.R., Nakafero G., Grainge M.J., Mallen C.D., Van-Tam J.S.N., Williams H.C., Abhishek A. (2023). Is vaccination against COVID-19 associated with inflammatory bowel disease flare? self-controlled case series analysis using the UK CPRD. Off. J. Am. Coll. Gastroenterol..

[63] Manser C.N., Paul M., Rogler G., Held L., Frei T. (2013). Heat waves, incidence of infectious gastroenteritis, and relapse rates of inflammatory bowel disease: A retrospective controlled observational study. Off. J. Am. Coll. Gastroenterol..

[64] Manser C.N., Kraus A., Frei T., Rogler G., Held L. (2017). The impact of cold spells on the incidence of infectious gastroenteritis and relapse rates of inflammatory bowel disease: A retrospective controlled observational study. Inflamm. Intest. Dis..

[65] Peng J.C., Ran Z.H., Shen J. (2015). Seasonal variation in onset and relapse of IBD and a model to predict the frequency of onset, relapse, and severity of IBD based on artificial neural network. Int. J. Color. Dis..

[66] Vavricka S.R., Rogler G., Maetzler S., Misselwitz B., Safroneeva E., Frei P., Manser C.N., Biedermann L., Fried M., Higgins P. (2014). High altitude journeys and flights are associated with an increased risk of flares in inflammatory bowel disease patients. J. Crohn’s Colitis.

[67] Park J., Yoon H., Shin C.M., Park Y.S., Kim N., Lee D.H. (2022). Clinical factors to predict flare-up in patients with inflammatory bowel disease during international air travel: A prospective study. PLoS ONE.

[68] Pagnini C., Menasci F., Festa S., Rizzatti G., Corleto V.D., Delle Fave M., D’Ambra G., Di Giulio E., Delle Fave G. (2015). Application of clinical indexes in ulcerative colitis patients in regular follow-up visit. correlation with endoscopic’mucosal healing’and implication for management. Eur. Rev. Med. Pharmacol. Sci..

[69] Gomollón F., Gisbert J.P., Guerra I., Plaza R., Villarroya R.P., Almazán L.M., Martín M.C.L., Antonaya M.D., Mendoza M.I.V., Aparicio J. (2022). Clinical characteristics and prognostic factors for Crohn’s disease relapses using natural language processing and machine learning: A pilot study. Eur. J. Gastroenterol. Hepatol..

[70] Swaminathan A., Fan D., Borichevsky G.M., Mules T.C., Hirschfeld E., Frampton C.M., Day A.S., Siegel C.A., Gearry R.B. (2022). The disease severity index for inflammatory bowel disease is associated with psychological symptoms and quality of life, and predicts a more complicated disease course. Aliment. Pharmacol. Ther..

[71] Hosseini S.V., Safarpour A.R., Taghavi S.A. (2015). Developing a novel risk-scoring system for predicting relapse in patients with ulcerative colitis: A prospective cohort study. Pak. J. Med. Sci..

[72] Stallmach A., Bokemeyer B., Helwig U., Lügering A., Teich N., Fischer I., Rath S., Lang D., Schmidt C., EPIC Study Group (2019). Predictive parameters for the clinical course of Crohn’s disease: Development of a simple and reliable risk model. Int. J. Color. Dis..

[73] Riggott C., Fairbrass K.M., Black C.J., Gracie D.J., Ford A.C. (2023). Novel symptom clusters predict disease impact and healthcare utilisation in inflammatory bowel disease: Prospective longitudinal follow-up study. Aliment. Pharmacol. Ther..

[74] Pallotta N., Vincoli G., Pezzotti P., Giovannone M., Gigliozzi A., Badiali D., Vernia P., Corazziari E.S. (2018). A risk score system to timely manage treatment in Crohn’s disease: A cohort study. BMC Gastroenterol..

[75] Dhingra R., Kedia S., Mouli V.P., Garg S.K., Singh N., Bopanna S., Singla V., Choudhury B.N., Verma P., Tiwari V. (2017). Evaluating clinical, dietary, and psychological risk factors for relapse of ulcerative colitis in clinical, endoscopic, and histological remission. J. Gastroenterol. Hepatol..

[76] Nakov R., Nakov V. (2021). Young age and short duration of the disease are associated with more frequent relapses in inflammatory bowel disease patients. Med. Pharm. Rep..

[77] Malibary N.H., Ezzat M.A., Mogharbel A.M., Kouzaba K.A., Alkadi A.A., Malki U.H., Gharib S.M., Altowairqi F.M., Saadah O.I., Mosli M.H. (2021). Factors affecting ulcerative colitis flare-ups: Associations with smoking habits and other patient characteristics. Cureus.

[78] Bischoff S.C., Escher J., Hébuterne X., Kłęk S., Krznaric Z., Schneider S., Shamir R., Stardelova K., Wierdsma N., Wiskin A.E. (2020). ESPEN practical guideline: Clinical Nutrition in inflammatory bowel disease. Clin. Nutr..

[79] Laing B.B., Lim A.G., Ferguson L.R. (2019). A Personalised Dietary Approach—A Way Forward to Manage Nutrient Deficiency, Effects of the Western Diet, and Food Intolerances in Inflammatory Bowel Disease. Nutrients.

[80] Guerreiro C.S., Ferreira P., Tavares L., Santos P.M., Neves M., Brito M., Cravo M. (2009). Fatty acids, IL6, and TNFα polymorphisms: An example of nutrigenetics in Crohn’s disease. Off. J. Am. Coll. Gastroenterol..

[81] Armstrong H.K., Bording-Jorgensen M., Santer D.M., Zhang Z., Valcheva R., Rieger A.M., Kim J.S.-H., Dijk S.I., Mahmood R., Ogungbola O. (2023). Unfermented β-fructan fibers fuel inflammation in select inflammatory bowel disease patients. Gastroenterology.

[82] Tanaka M., Iwao Y., Sasaki S., Okamoto S., Ogata H., Hibi T., Kazuma K. (2007). Moderate dietary temperance effectively prevents relapse of Crohn disease: A prospective study of patients in remission. Gastroenterol. Nurs..

[83] World Health Organization. Regional Office for the Eastern Mediterranean. (2019). Healthy Diet.

[84] Lichtenstein G.R., Loftus E.V., Isaacs K.L., Regueiro M.D., Gerson L.B., Sands B.E. (2018). ACG clinical guideline: Management of Crohn’s disease in adults. Off. J. Am. Coll. Gastroenterol..

[85] To N., Ford A.C., Gracie D.J. (2016). Systematic review with meta-analysis: The effect of tobacco smoking on the natural history of ulcerative colitis. Aliment. Pharmacol. Ther..

[86] Lamers C.R., De Roos N.M., Heerink H.H., Van de Worp-Kalter L.A., Witteman B.J. (2022). Lower impact of disease on daily life and less fatigue in patients with inflammatory bowel disease following a lifestyle intervention. Inflamm. Bowel Dis..

[87] Singh S., Graff L.A., Bernstein C.N. (2009). Do NSAIDs, antibiotics, infections, or stress trigger flares in IBD?. Off. J. Am. Coll. Gastroenterol..

[88] Govani S.M., Wiitala W.L., Stidham R.W., Saini S.D., Hou J.K., Feagins L.A., Sussman J.B., Higgins P.D., Waljee A.K. (2016). Age disparities in the use of steroid-sparing therapy for inflammatory bowel disease. Inflamm. Bowel Dis..

[89] Asscher V.E., Waars S.N., van der Meulen-de A.E., Stuyt R.J., Baven-Pronk A.M.C., van Der Marel S., Jacobs R.J., Haans J.J., Meijer L.J., Klijnsma-Slagboom J.D. (2022). Deficits in geriatric assessment associate with disease activity and burden in older patients with inflammatory bowel disease. Clin. Gastroenterol. Hepatol..

[90] European Crohn’s and Colitis Organisation Phychosocial Issues. https://www.e-guide.ecco-ibd.eu/interventions-investigational/psychosocial-issues.

[91] Olivera P., Danese S., Jay N., Natoli G., Peyrin-Biroulet L. (2019). Big data in IBD: A look into the future. Nat. Rev. Gastroenterol. Hepatol..

[92] Borg-Bartolo S.P., Boyapati R.K., Satsangi J., Kalla R. (2020). Precision medicine in inflammatory bowel disease: Concept, progress and challenges. F1000Research.

[93] La Rosa G.R.M., Lorenzo-Pouso A.I., Caponio V.C.A., Puci M.V. (2025). Apical periodontitis in inflammatory bowel disease: A meta-analysis at patient and tooth level. Front. Dent. Med..

[94] Wang Q., Chen S., Zhou J., Zhao L. (2024). Bidirectional associations between periodontitis and inflammatory bowel disease: A systematic review of longitudinal studies with meta-analysis and trial sequential analysis. J. Periodontal Res..

[95] Kumar A., Teslova T., Taub E., Miller J.D., Lukin D.J. (2021). Comorbid diabetes in inflammatory bowel disease predicts adverse disease-related outcomes and infectious complications. Dig. Dis. Sci..

